# Inhibition of Proliferation and Induction of Autophagy by Atorvastatin in PC3 Prostate Cancer Cells Correlate with Downregulation of Bcl2 and Upregulation of miR-182 and p21

**DOI:** 10.1371/journal.pone.0070442

**Published:** 2013-08-01

**Authors:** Xinjian Peng, Wenping Li, Liang Yuan, Rajendra G. Mehta, Levy Kopelovich, David L. McCormick

**Affiliations:** 1 Life Sciences Group, IIT Research Institute, Chicago, Illinois, United States of America; 2 Chemopreventive Agent Development Research Group, Division of Cancer Prevention, National Cancer Institute, Bethesda, Maryland, United States of America; University of Kentucky College of Medicine, United States of America

## Abstract

The epidemiologic association between statin use and decreased risk of advanced prostate cancer suggests that statins may inhibit prostate cancer development and/or progression. Studies were performed to determine the effects of a model statin, atorvastatin (ATO), on the proliferation and differentiation of prostate cancer cells, and to identify possible mechanisms of ATO action. ATO inhibited the *in vitro* proliferation of both LNCaP and PC3 human prostate cancer cells in a dose- and time-dependent fashion. The greater inhibitory activity of ATO in PC3 cells was associated with induction of autophagy in that cell line, as demonstrated by increased expression of LC3-II. miR-182 was consistently upregulated by ATO in PC3 cells, but not in LNCaP cells. ATO upregulation of miR-182 in PC3 cells was p53-independent and was reversed by geranylgeraniol. Transfection of miR-182 inhibitors decreased expression of miR-182 by >98% and attenuated the antiproliferative activity of ATO. miR-182 expression in PC3 cells was also increased in response to stress induced by serum withdrawal, suggesting that miR-182 upregulation can occur due to nutritional stress. Bcl2 and p21 were identified to be potential target genes of miR-182 in PC3 cells. Bcl2 was downregulated and p21 was upregulated in PC3 cells exposed to ATO. These data suggest that miR-182 may be a stress-responsive miRNA that mediates ATO action in prostate cancer cells.

## Introduction

Statins are used widely for the prevention and treatment of hypercholesterolemia; the cholesterol lowering activity of statins is effected through their inhibition of 3-hydroxyl-3-methyl-glutaryl coenzyme A (HMG-CoA) reductase, a key enzyme in cholesterol biosynthesis [1], [2]. In addition to effects on cholesterol biosynthesis, statins such as atorvastatin (ATO) have attracted considerable interest for their possible utility for cancer prevention and therapy [3], [4].

The results of several epidemiology studies and meta-analyses suggest an inverse relationship between statin use and prostate cancer risk, especially the risk of advanced or metastatic prostate cancer [5], [6], [7]. Recent data from studies in experimental prostate cancer models demonstrate that co-administration of statins with other agents can yield additive or synergistic anticancer effects [4], [8]. Several potential mechanisms have been identified through which statins may modulate cancer progression; these mechanisms include inhibition of cell proliferation, induction of autophagy and apoptosis, and inhibition of angiogenesis [3], [9], [10]. Statins are potent inhibitors of mevalonate biosynthesis [11], resulting in the inhibition of protein prenylation; the antiproliferative and anticancer effects of statins could be affected through this pathway. However, the specific biochemical mechanism(s) through which ATO and other statins exert cancer preventive and/or therapeutic activity in the prostate remain largely undefined.

Autophagy is a cellular process through which macromolecules and organelles are degraded during periods of cellular stress associated with nutrient depletion, infection, or apoptosis [9]. Recent *in vitro* data demonstrate that ATO can induce autophagy and autophagy-associated cell death in PC3 prostate cancer cells [9]. On this basis, the induction of autophagy provides a potential mechanism through which the inhibition of prostate cancer progression by ATO may be effected. In PC3 prostate cancer cells, ATO induces autophagic flux, cell cycle arrest and then cell death [9]. In this process, induction of autophagy appears to be a necessary step prior to cell death [9], [12].

miRNAs are small non-coding RNAs that control gene expression by triggering translation repression or degradation of mRNA [13], [14]. miRNAs appear to be involved in the regulation of a broad range of cellular processes, and altered patterns of miRNA expression are seen in a number of pathologic conditions. Accumulating evidence suggests that miRNA expression is altered in cancers in several sites, including the prostate [15], [16], [17]; alterations in the expression of specific miRNAs could provide a mechanism through which pharmacologic agents and dietary manipulations may inhibit cancer induction and/or progression. In addition, miRNA profiling may be useful in characterizing molecular signatures of neoplasms [18] and in identifying potential targets for the development of anticancer drugs [19]. For example, expression of miRNAs in cancer cells is modulated by cancer therapeutics such as doxorubicin and trastuzumab [20], [21]. Similarly, the modulation of miRNA expression by dietary components such as folate, retinoids, and curcumin may underlie their cancer preventive activities [18]. On this basis, we hypothesized that alterations in miRNA expression could be responsible for, or associated with, ATO action in prostate cancer cells, and that altered expression of specific miRNAs is linked to the induction of autophagy by ATO.

## Materials and Methods

### Reagents

Atorvastatin (ATO) was supplied by the Chemopreventive Agent Repository maintained by the Division of Cancer Prevention, National Cancer Institute, Rockville, MD. ATO was dissolved in DMSO (50 mM) and was stored at −20°C prior to use. Geranylgeraniol (GGOH), farnesol (FOH), CoQ10, squalene, and MTT (3-(4,5-dimethylthiazol-2-yl)-2,5-diphenyltetrazolium bromide) were purchased from Sigma-Aldrich (St. Louis, MO).

### Cell Lines, Cell Culture and Cell Proliferation Assay

Human prostate cancer cells (PC3 cells and LNCaP cells) were purchased from the American Type Culture Collection (Manassas, VA). Sublines of PC3 cells (clones 7, 19 and 20) that were established through clonal selection [22] were kindly provided by Dr. Ann R. Kennedy at the University of Pennsylvania. All prostate cancer cell lines were cultured in RPMI medium containing 10% FBS. Cell proliferation was quantitated using the MTT assay or crystal violet (CV) assay, or through direct cell counting using a Z2 Coulter Particle Counter (Beckman Coulter, Brea, CA). Preliminary studies performed in our laboratory have demonstrated that the absorbance (OD value) for both MTT and CV assays is proportional to cell number.

### Western Blot Analysis

At 40–50% confluence, cells were exposed to ATO for 24 h, 48 h or 72 h. After exposure, cell lysates were prepared and 50 µg total protein was loaded for western blot analysis. LC3-II polyclonal antibody was purchased from Abgent (San Diego, CA). Bcl2 and p21 mouse monoclonal antibodies were from NeoMarker (Fremont, CA). Actin polyclonal antibody and α-tubulin, p53 and PCNA monoclonal antibodies and all secondary antibodies were purchased from Santa Cruz Biotech (Santa Cruz, CA).

### Microarray Analysis

PC3 and LNCaP cells were seeded in culture dishes so that cells reached ∼30% confluence on Day 2. At that time, culture medium was replaced and cells were exposed to ATO (10 µM) for 24 h. Total RNA to be used in both mRNA and miRNA assays was isolated and purified using the Ribopure RNA isolation kit (Invitrogen, Grand Island, NY). mRNA microarray and miRNA array profiling were performed by GenUS BioSystem (Northbrook, IL) using Agilent Human v2 GE 4x44K arrays and Agilent Human miRNA v14 Rev.2 arrays, respectively; parallel microarray and miRNA profiling were performed using the same total RNA samples. Data were analyzed using the Agilent Feature Extraction and GeneSpring GX v7.3.1 software packages. Microarray files for the 4 samples analyzed in this report are available on GEO, accession GSE46376.

### Quantitative RT-PCR

Total RNA was extracted with trizol reagent (Invitrogen), and mRNA analyses were performed as described previously [23]. Two RT reactions for each sample were pooled and diluted with an equal amount of DNase/RNase free water. Real-time PCR was performed with 2 µL diluted RT product in a MyiQ Real-Time PCR Detection System (Bio-Rad, Hercules, CA) using iQ™ SYBR Green PCR Supermix (Bio-Rad) according to the manufacturer’s instructions [24]. Gene-specific primers were designed with Primer 3 (http://frodo.wi.mit.edu/cgi-bin/primer3/primer3_www.cgi). Actin was used as a reference gene for normalization of data. Fold inductions were calculated using the formula 2^−(ΔΔCt)^, where ΔΔCt is ΔCt_(treatment)_ – ΔCt_(control)_, ΔCt is Ct_(target gene)_ – Ct_(actin)_ and Ct is the cycle at which the threshold is crossed.

For miRNA analysis, 20 ng of total RNA was used for cDNA synthesis by the Taqman MicroRNA Reverse Transcription kit (Invitrogen); miRNA expression levels were quantified using the Taqman MicroRNA assay (Invitrogen) by following the manufacturer’s instructions. Relative expression levels of miRNA were normalized to U6 snRNA and calculated as previously reported [25].

### miRNA Transfection

miRNA transfections were conducted in PC3 cells using lipofectomine 2000 (Invitrogen) following the manufacturer’s protocol. PC3 cells were cultured overnight in normal growth medium, then transfected with miR-182 mimic (Sigma) or miR-182 inhibitor or negative control in OPTI-MEM (Invitrogen) containing 3% FBS for 18–28 h. The miRNA mimic, inhibitor for miR-182, and negative control (Invitrogen) were each used at a final concentration of 20 nM. Transfected cells were then cultured in normal growth medium with or without ATO treatment for different time periods.

### Statistical Analysis

Normally distributed parametric data are presented as Mean ± SD, and were analyzed by Student’s t-test or one-way ANOVA; *post-hoc* analyses were performed using Tukey’s Multiple Comparison Test. Statistical analyses were performed using GraphPad Software (San Diego, CA) and Microsoft Office Excel. Differences between means were considered to be significant at p<0.05.

## Results

### Differential Effects of Atorvastatin in LNCaP and PC3 Prostate Cancer Cells

**Figure 1:**
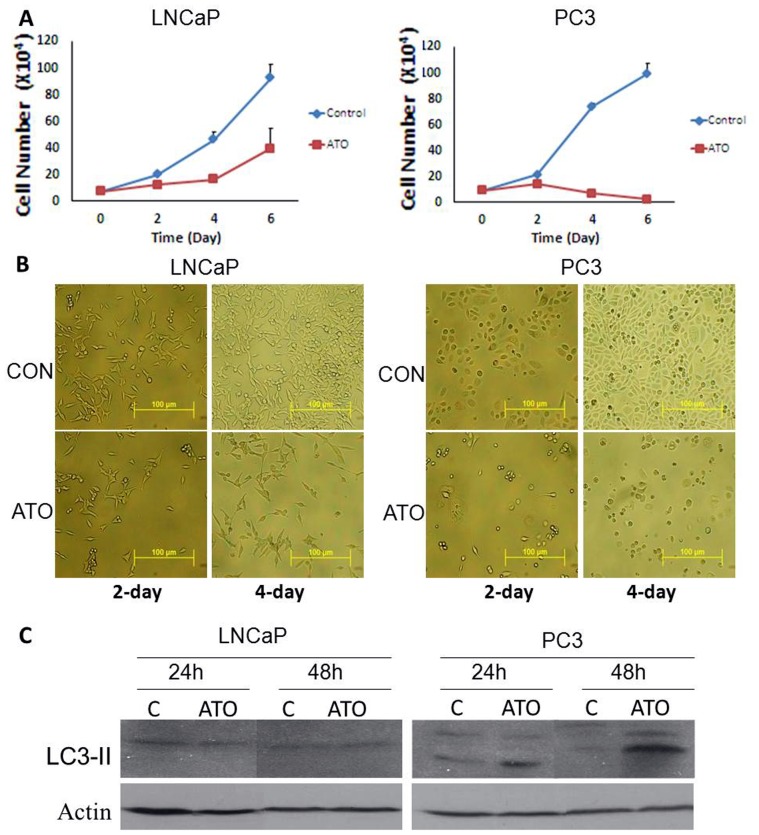
Effect of ATO on cell proliferation, morphology and autophagy marker LC3-II expression. (A) LNCaP and PC3 cells were seeded in 6 well plates (36000 cells/well) and incubated overnight and then treated with DMSO (control) or 10 µM ATO for 2, 4 and 6 days. Cell numbers are expressed as Mean ± SD, n = 3. (B) Microscopy of LNCaP and PC3 cells after treatment with10 µM ATO for 2 and 4 days. C: LNCaP and PC3 cells were treated with 5 µM ATO for 24 and 48 h, cell lysates were collected for LC3-II expression analysis using western blot. ATO induced LC3-II expression only in PC3 cells.

When administered at concentrations ranging from 0–20 µM, ATO inhibited cell proliferation in both LNCaP and PC3 cells in a dose- and time-dependent manner; the lowest effective dose in both cell lines was 2.5 µM (data not shown). Representative growth curves for LNCaP and PC3 cells cultured in the absence and presence of ATO (10 µM) are provided in Fig. 1A. At a concentration of 10 µM, ATO induced a continuous, time-dependent inhibition of LNCaP cell proliferation over the 6-day exposure period; no evidence of cell death was seen in LNCaP cells exposed to ATO at 10 µM for this period. In PC3 cells, ATO inhibition of cell proliferation was seen on Day 2; substantial cell death in PC3 cells was seen on Days 4 and 6, as cell counts in ATO-treated groups at these times were below the number of cells originally seeded. These data suggest that (a) PC3 cells are more sensitive to ATO than are LNCaP cells, and (b) the mechanisms of action of ATO in the two prostate cancer cell lines may be different.

The effects of ATO (10 µM) on cell density and cellular morphology in LNCaP and PC3 cells after two and four days of exposure are shown in Figure 1B. Morphological alterations were present in both LNCaP and PC3 cells exposed to ATO; cell density changes were consistent with the changes in cell numbers reported above. In the process of autophagosome formation, cytosolic microtubule-associated protein light chain 3 (LC3) is conjugated with phosphatidylethanolamine (PE) at its carboxyl terminus [26], [27]. The conjugated LC3 (identified as LC3-II) is then inserted into autophagic vesicle membrane. LC3-II can be detected by immunoblot or immunostaining, and is widely used as a biochemical marker for cellular autophagy [28], [29]. Using LC3-II as a biomarker of autophagy, western blot analyses demonstrated that ATO induced autophagy in PC3 cells, but not in LNCaP cells (Fig. 1C). These results link the induction of autophagy by ATO in PC3 prostate cancer cells with cell death in these cells; neither autophagy nor cell death were induced by ATO in LNCaP cells.

### Effects of Atorvastatin on Cell Proliferation and LC3-II Expression in PC3 Cells

**Figure 2:**
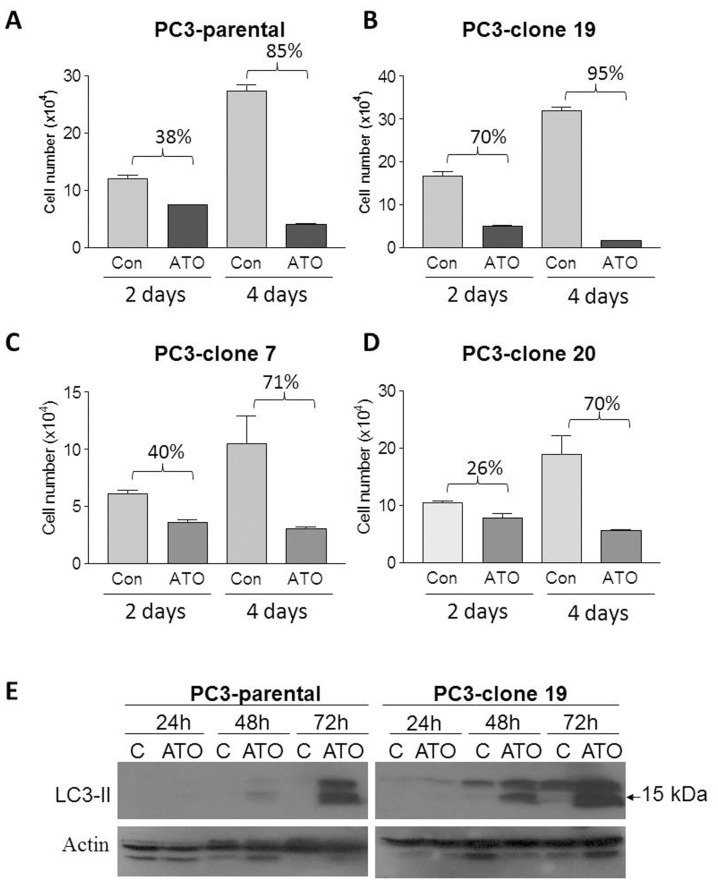
Cellular sensitivity in response to ATO correlates to autophagy induction in PC3 cells. (A–D) PC3 parental (A) and PC3 clone 19 (B), 7(C) and 20 (D) cells were cultured in 6 well plates and treated with 5 µM ATO for 2 or 4 days. Cell numbers are expressed as Mean ± SD, n = 3. (C) PC3 parental and PC3-clone 19 cells were treated with 5 µM ATO side by side with the proliferation assay (A & B) for 24 h, 48 h or 72 h. Cell lysates were collected for western blot analysis of LC3-II expression.

To confirm the association between autophagy and cell death in PC3 prostate cancer cells exposed to ATO, we first characterized the effects of ATO on cell counts in parental PC3 cells and several PC3 sublines [22]. Exposure of parental PC3 cells to ATO (5 µM) inhibited cell proliferation by 38% on Day 2 (p<0.01) and 85% (p<0.001) on Day 4 (Fig. 2A). All the sublines (clone 19, 7, and 20) (Fig. 2B–D) were responsive to ATO treatment, but PC3 clone 19 was most sensitive to ATO among the three tested clones: after two and four days of exposure, ATO (5 µM) inhibited the proliferation of PC3 clone 19 cells by 70% and 95%, respectively (p<0.001 for both comparisons; Fig. 2B); whereas other two clones (clone 7 and 20) responded to ATO in a similar fashion as parental cells with some variability. Besides, significant cell death was observed in all the cell lines on Day 4. Therefore parental and the most sensitive clone were selected for further characterization of LC3-II expression. In both PC3 parental and PC3-clone 19 cells, ATO induced LC3-II expression in a time-dependent manner (Fig. 2E); however, in the sensitive PC3-clone 19 cells, LC3-II demonstrated more rapid and quantitatively greater induction by ATO. These data suggest that ATO induced autophagy activity correlates with cellular sensitivity in response to ATO, and that autophagy may be involved in cell death induced by ATO in PC3 cells.

### Effects of Atorvastatin on Gene Expression in LNCaP and PC3 Cells (Microarray Studies and Pathway Analysis)

Microarray analyses were performed to determine the effects of ATO on gene expression and miRNA expression in LNCaP and PC3 cells; side by side comparisons were performed using total RNA samples isolated from cells treated with ATO (10 µ M) for 24 h. ATO induced statistically significant changes in the expression of 1427 genes in LNCaP cells and 3755 genes in PC3 parental cells (Fig. S1). KEGG pathways analysis of the 1427 differentially expressed genes in LNCaP cells identified 7 functional pathways for which the differential expression of component genes was statistically significant at *p*≤0.01 (Table S1). Some pathways (*e.g*., cholesterol biosynthesis, steroid biosynthesis) were predictable on the basis of the known activity of ATO as an inhibitor of HMG Co-A reductase; others may be specifically related to the antiproliferative activity of ATO in LNCaP cells. As might be expected, a much larger number of altered functional pathways (46 pathways) was identified through KEGG analysis of differentially expressed genes in PC3 cells exposed to ATO (Table S2). In addition to pathways that overlapped those identified in LNCaP cells, ATO modulated several pathways with clear links to cell proliferation and cell death; these functional pathways included DNA replication, cell cycle, and MAPK signaling.

### Microarray Studies Of Atorvastatin And Mirna Expression In Lncap And Pc3 Cells

ATO induced differential expression of numerous miRNAs in LNCaP and PC3 cells. Using cutoff values of 1.5 for fold-change differences in expression and p<0.05 for statistical significance, 131 miRNAs were differentially expressed in LNCaP cells exposed to ATO, and 111 miRNAs were differentially expressed in PC3 cells exposed to ATO (Fig. S1).

To identify miRNAs whose differential expression could underlie ATO cytotoxicity in PC3 cells, a fold change cutoff of ≥2.00 was applied to PC3 data, followed by removal of miRNAs whose directional changes in expression were the same in PC3 and LNCaP cells. This strategy identified a total of 42 miRNAs that were differentially regulated by ATO specifically in PC3 cells (Table S3).

### Mir-182 Is Up-Regulated By Ato In Pc3 Cells

To confirm the miRNA array data in PC3 cells, qRT-PCR (Taqman technique) was used to confirm the differential expression of three miRNAs (miR-555, miR-654 and miR-182) identified as up-regulated by ATO and three miRNAs (miR-494, miR-1255a and miR-550a) that were down-regulated by ATO. These specific miRNAs were selected for further study on the basis of fold-change differences in expression seen in microarray studies. miR-182 (which was up-regulated by four-fold in PC3 cells exposed to ATO) was also selected due to its known relationship to cell stress [29].

**Figure 3:**
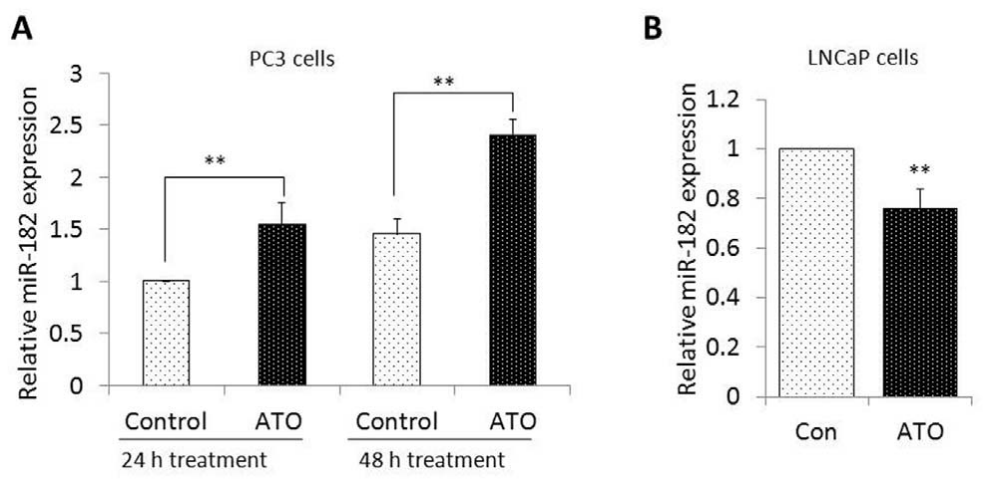
Effect of ATO on miR-182 expression in prostate cancer cells. (A) PC3 cells were exposed to 5 µM ATO for 24 h and 48 h. Total RNA was isolated and subjected to Taqman qRT-PCR analysis using primer sets for miR-182. (B) LNCaP cells were exposed to 5 µM ATO for 48 h, followed by analysis of miR-182 expression. Data are summarized from 3 independent experiments and expressed as Mean ± SD. **p<0.01.

ATO up-regulation of miR-182 in PC3 cells was confirmed by qRT-PCR (Fig. 3A); other miRNAs were found to be expressed only at very low levels, and expression levels of these miRNAs demonstrated substantial variability between samples. miR-182 expression was increased by 56% (p<0.01) at 24 h and 66% (p<0.01) at 48 hours in PC3 cells exposed to ATO (Fig. 3A). By contrast, ATO down-regulated miR-182 in LNCaP cells by 24% (p<0.01) at 48 hrs (Fig. 3B). These data suggest that miR-182 regulation by ATO in prostate cancer cells is cell-specific, and that up-regulation of miR-182 may be involved in ATO action in PC3 cells.

### Mir-182 Upregulation By Ato In Pc3 Cells Is Reversed By Geranylgeraniol Co-Treatment And Is Independent Of P53 Expression

**Figure 4:**
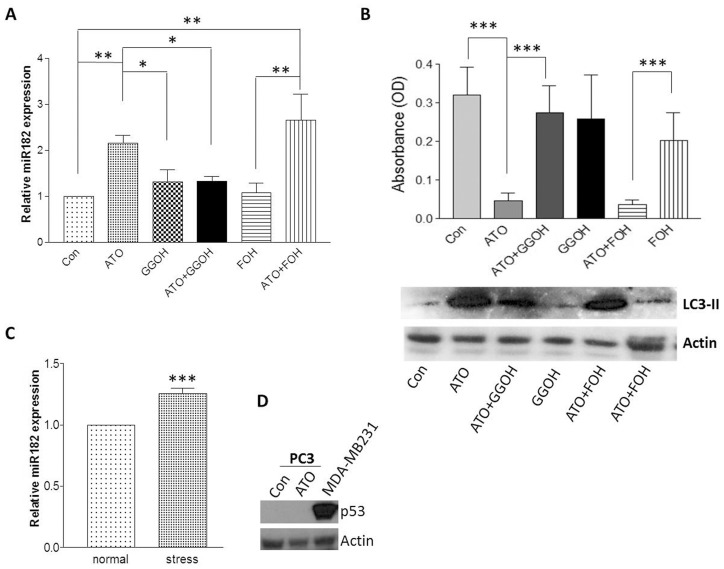
Geranylgeraniol co-treatment reversed ATO effect on miR-182 expression. (A) PC3 cells were treated with 5 µM ATO for 48 h in the presence and absence of metabolites including geranylgeraniol (10 µM) and farnesol (10 µM), miR182 expression was then detected by qRT-PCR. (B) PC3 cells were treated with 5 µM ATO for 2 or 4 days in the presence and absence of geranylgeraniol (10 µM) and farnesol (10 µM), cell proliferation (4 day treatment) was evaluated by MTT assay and LC3-II expression (2 day treatment) was examined by western blot. (C) PC3 cells were cultured in normal growth medium (10% FBS) overnight, and were then stressed by culturing in low serum medium (1% FBS) for 48 h, followed by quantitation of miR-182 expression by qRT-PCR. (D) p53 expression was examined by western blot analysis in PC3 cells, MDA-MB-231 breast cancer cells served as a positive control for p53 expression. For all bar graphs, data are expressed as Mean ± SD; for qRT-PCR analyses, n = 3; for MTT assay, n = 8; *p<0.05, **p<0.01, ***p<0.001.

We previously reported that miR-182 is a stress-responsive miRNA in breast epithelial cells [29]. Since miR-182 is upregulated by ATO, and ATO can cause cellular stress by inhibiting mevalonate biosynthesis [3],[9], we hypothesized that miR-182 upregulation by ATO might be due to ATO-induced cellular stress. If this hypothesis is correct, removal of ATO stress would reverse miR-182 up-regulation. To evaluate this hypothesis, miR-182 expression was quantitated in PC3 cells treated with ATO ± several mevalonate metabolites whose synthesis is inhibited as a result of ATO suppression of mevalonate biosynthesis. Agents studied included geranylgeraniol (GGOH), farnesol (FOH), co-enzyme Q10 (coQ10), and squalene. In a preliminary study, only GGOH reversed the effects of ATO on miR-182 expression in PC3 cells. On this basis, a definitive series of experiments was performed using GGOH and FOH. Consistent with the results of the preliminary study, GGOH reversed the effect of ATO on miR-182 expression; FOH had no effect (Fig. 4A). We also evaluated the effect of GGOH and FOH on ATO-mediated inhibition of cell proliferation and induction of autophagy in PC3 cells. As shown in Fig. 4B, GGOH also reversed the effect of ATO on cell proliferation and LC3-II expression; FOH had no effect. These data clearly indicate that ATO-mediated inhibition of biosynthesis of geranylgeraniol, but not farnesol, results in upregulation of miR-182, suppression of proliferation and induction of autophagy.

We used a second stress model – serum deprivation – to determine the specificity of miR-182 responses to ATO and to determine whether miR-182 expression is also be enhanced by other types of stress in PC3 cells. PC3 cells were cultured in growth medium containing 10% FBS, followed by culturing for two days in growth medium containing only 1% FBS. Serum deprivation for 2 days increased miR-182 expression by 26% (p<0.001; Fig. 4C), suggesting that miR-182 up-regulation in PC3 cells is induced by different types of stressors.

p53 is considered to be tightly associated with cell stress [30]. Because regulation of miR-182 has recently been reported to be p53-dependent [20], we examined levels of p53 expression in PC3 cells with or without exposure to ATO. p53 protein is not expressed in PC3 cells under either condition (Fig. 4D), demonstrating that miR-182 regulation by ATO in PC3 cells is p53-independent.

### Ato Regulates Bcl2 And P21, Which Are Potential Targets Of Mir-182 In Pc3 Cells

The effects of ATO on potential target genes of miR-182 were studied in order to evaluate the hypothesis that ATO modulation of miR-182 expression induces changes in regulatory function. To address this hypothesis, TargetScan (http://www.targetscan.org/) and PicTar (http://pictar.mdc-berlin.de/) were used to identify potential target genes of miR-182; genes identified using these computer simulation programs were then screened using microarray data. Through this iterative process, 41 potential target genes of miR-182 were identified; 24 genes were upregulated and 17 genes were downregulated by ATO (Table S4).

**Figure 5:**
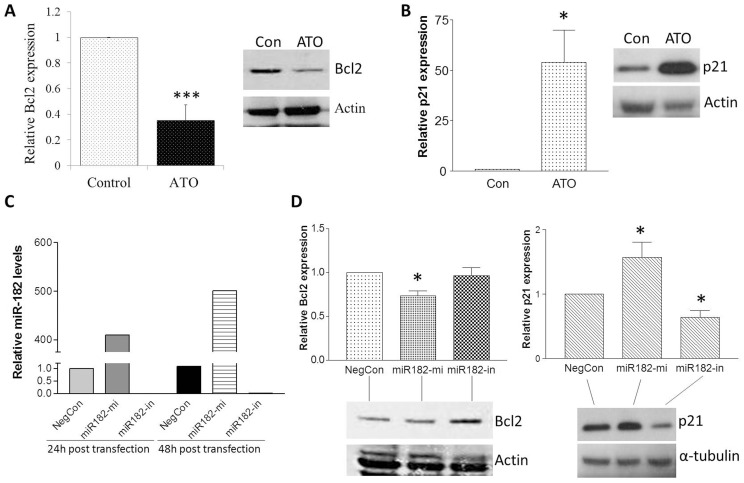
Effect of ATO and alteration of miR-182 on expression of Bcl2 and p21. (A) & (B) PC3 cells were treated with 5 µM ATO for 48 h, followed by qRT-PCR and western blot analysis of Bcl2 (A) and p21(B). Data are expressed as Mean ± SD. n = 3. ATO decreased both mRNA and protein expression of Bcl2 whereas increased both mRNA and protein expression of p21. (C) & (D) PC3 cells were transfected with a negative control (NegCon), miR-182 mimic (miR182-mi), or miR-182 inhibitor (miR182-in) for 24 and/or 48 h, followed by analysis of expression of miR-182 (C), Bcl2 and p21(D). C confirms the successful transfection of miR182-mi and miR182-in by detection of increased or decreased expression of miR-182. D demonstrates Bcl2 and p21 expression status at mRNA (top) and protein levels (bottom) at 48 h post transfection. miR-182 overexpression decreased Bcl2 mRNA expression, but had minimal effect on its protein expression; while miR-182 knock-down had no effect on Bcl2 mRNA expression, but increased its protein expression. p21 was positively correlated to miR-182 expression at both mRNA and protein levels. Actin or α-tubulin served as loading controls for protein expression.

Because miR-182 was upregulated by ATO, the direct targets of miR-182 are most likely to be downregulated. Using differential gene expression cutoff values of ≥2.0 with statistical significance set at p≤0.05, we identified five miR-182 target genes that were down-regulated by ATO in PC3 cells; genes identified through this process were Bcl2, BNC2, FRMD4A, ELL and AMOTL2. qRT-PCR analyses confirmed that Bcl2 and FRMD4A are each downregulated by 65% in PC3 cells exposed to ATO (p<0.001 for both genes; data for Bcl2 are shown in Fig. 5A). Down-regulation of Bcl2 by ATO was also confirmed at the protein level (Fig. 5A).

We also found that p21 CDK inhibitor, an important negative regulator of the cell cycle, is consistently up-regulated in PC3 cells exposed to ATO, up-regulation of p21 by ATO was demonstrated at both the mRNA and protein levels (Fig. 5B).

A series of transfection studies was performed to elucidate the relationship between ATO exposure, miR-182 expression, and Bcl2 and p21 mRNA and protein expression in PC3 cells. As shown in Fig. 5C, transfection of miR-182 mimic increased miR-182 expression by 400–500 fold at both 24 h and 48 h after transfection; by contrast, transfection of miR-182 inhibitor suppressed miR-182 expression by >98%. Overexpression of miR-182 decreased Bcl2 mRNA levels by 26% (p<0.05), but had no significant effect on Bcl2 protein expression at 48 hours. Transfection of miR-182 inhibitor had no effect on Bcl2 mRNA expression, but did significantly increase Bcl2 protein expression (Fig. 5D). These data suggest that Bcl2 is a direct target of miR-182 in PC3 cells; since the basal protein level of Bcl2 was already low, there was little protein expression available to demonstrate further down-regulation through miR-182 overexpression. These results also suggest that miR-182 may not contribute to Bcl2 down-regulation in response to ATO in PC3 cells.

By contrast, p21 was not identified as a direct target of miR-182 by either TargetScan or PicTar. Interestingly, p21 expression was positively correlated with miR-182 expression at both the mRNA and protein levels (Fig. 5D). Overexpression of miR-182 increased p21 mRNA expression by 57% (p<0.05), while knock-down of miR-182 by transfecting miR-182 inhibitor decreased p21 mRNA expression by 36% (p<0.05). These data suggest that p21 is an indirect target of miR-182, and that miR-182 may contribute in some manner to p21 up-regulation by ATO in PC3 cells.

### Effect Of Mir-182 Expression On Cell Proliferation In Response To Ato In Pc 3 Cells

**Figure 6:**
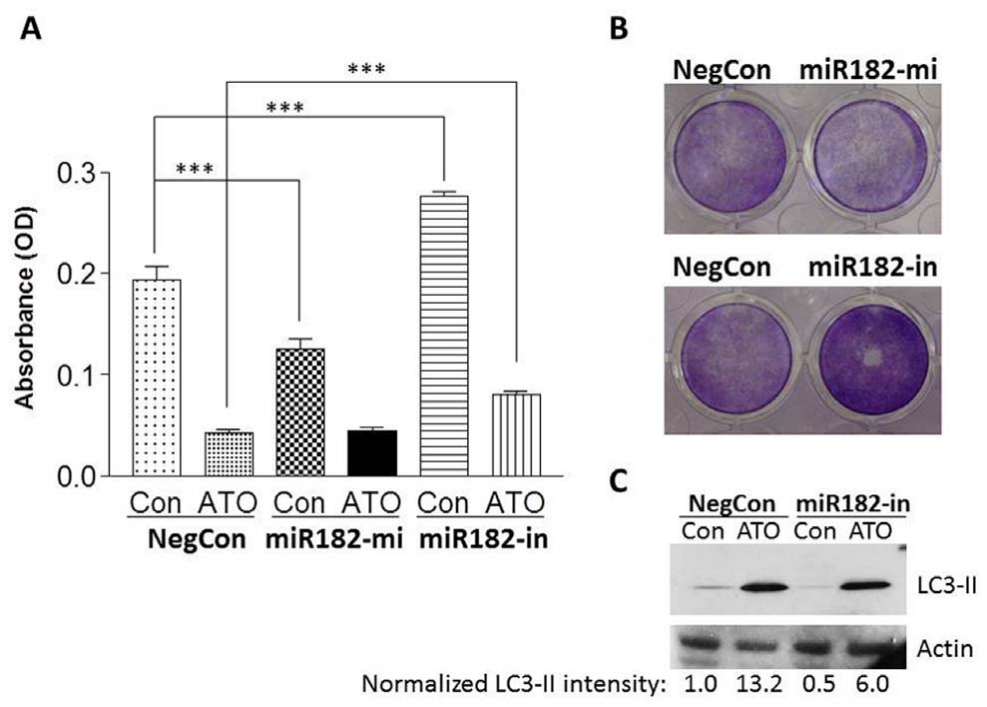
Functional significance of miR-182 in ATO induced suppression of PC3 cell proliferation and induction of autophagy. (A) PC3 cells were transfected with miR-182 mimic (miR182-mi, 20 nM) and miR-182 inhibitor (miR182-in, 20 nM) in 48 well plates respectively with a miRNA mimic that confirmed to have minimal sequence identity with miRNAs in human as a negative control (NegCon, 20 nM) and then treated with 2.5 µM ATO for 4 days, cell proliferation was evaluated by MTT assay. Absorbance values are expressed as Mean ± SD, ***p<0.001, n = 8. (B) PC3 cells were transfected as described in A, and cultured for another 4 days post transfection, cells were then stained with crystal violet using standard CV assay procedure; images of cell proliferation status were recorded with microscopy. (C) PC3 cells were transfected with a negative control or miR-182 inhibitor, then treated with 2.5 µM ATO for 48 h, cells were collected for western blot analysis of LC3-II expression. Actin served as a loading control. A representative western blot is shown. The values below each band represent the normalized LC3-II intensity (total pixels) of the presented western blot as determined by UN-SCAN-IT software (Silk Scientific Inc., Orem, UT).

To examine the functional role of miR-182 as a mediator of ATO responses, miR-182 expression was manipulated by transfecting miR-182 mimic or miR-182 inhibitor into PC3 cells. Transfected cells were exposed to a low concentration of ATO (2.5 µM) for 4 days, followed by quantitation of cell proliferation by MTT (Fig. 6A) or CV assay (Fig. 6B). The 2.5 µM concentration of ATO was used in order to permit the study of differential responses resulting from manipulation of miR-182 expression. As shown in Fig. 6A, overexpression of miR-182 in untreated cells inhibited proliferation by 36% (p<0.001); knock-down of miR-182 in otherwise untreated cells increased proliferation by 43% (p<0.001). miR-182 overexpression did not alter PC3 cell responses to ATO; however, knock-down of miR-182 reduced the activity of ATO as an inhibitor of cell proliferation (Fig. 6A). In the presence of ATO, the absorbance generated by miR-182 inhibitor-transfected cells increased by 89% (p<0.001, n = 8) in comparison to negative control-transfected cells. Fig. 6B shows the images of CV staining at 4 days after transfection of miR-182 mimic and miR-182 inhibitor in PC3 cells. The CV assay demonstrated similar results (data not shown) as MTT assay. These results demonstrate that miR-182 directly inhibits the proliferation of PC3 cells, and may also mediate the suppression of cell proliferation induced by ATO.

Since ATO induces autophagy in PC3 cells, we used western blots and UN-SCAN-IT software to determine the effects of miR-182 on LC3-II expression. Transfection with miR-182 inhibitor (miR-182-in) decreased the basal expression of LC3-II by ∼30% (n = 3, a representative western blot is shown in Fig. 6C). Whereas ATO induced LC3-II expression by ∼8.7 fold in PC3 cells transfected with negative control (NegCon), ATO induced LC3-II expression by only ∼6.3 fold in PC3 cells transfected with miR-182 inhibitor. When considered as a whole, these data suggest that miR-182 is involved in regulation of both PC3 cell proliferation and the induction of autophagy in PC3 cells.

## Discussion

Although an expanding body of evidence suggests that statins may have useful activity in prostate cancer prevention and/or therapy, the molecular mechanism(s) through which statins act to modulate neoplastic development and progression in the prostate are poorly understood. The goal of the present series of studies was to identify potential mechanisms of ATO action in prostate cancer cells, with specific emphasis on the possible role of miRNAs in statin activity.

PC3 cells are more sensitive to ATO than are LNCaP cells: in parallel studies using a range of ATO concentrations between 0 and 20 µ M, greater antiproliferative activity was seen in PC3 cells than in LNCaP cells. Furthermore, cell death was induced by ATO in PC3 cells only. Cell death in PC3 cells was correlated with induction of autophagy by ATO; this response was not seen in LNCaP cells. In comparisons of ATO responses in parental PC3 cells and PC3 subclones, cellular sensitivity to ATO was positively correlated with the induction of autophagy. These data suggest that induction of autophagy is an important determinant of prostate cancer cell sensitivity in response to ATO, and may provide a useful biomarker for responses of prostate cancer cells to therapeutic intervention.

Following demonstration of differential ATO activity in the inhibition of proliferation and the induction of autophagy in PC3 and LNCaP human prostate cancer cells, mRNA microarray and miRNA array studies were performed to identify miRNAs and target genes that may be involved in ATO action. A stress responsive miRNA, miR-182, was found to be up-regulated by ATO in PC3 cells; alterations in miR-182 levels were closely linked to the antiproliferative activities of ATO in these cells. Knock-down of miR-182 in PC3 cells was inversely associated with the expression of Bcl2 at protein levels; by contrast, upregulation of miR-182 was positively correlated to levels of p21 transcripts and protein.

Unexpectedly, the differential expression of several miRNAs identified in miRNA array studies could not be verified using Taqman qRT-PCR. Although the reason for this discrepancy is unknown, the apparently differential responses could reflect the presence of isomiRs [31], or miRNA variants, that are detectable by miRNA assays but not by Taqman qRT-PCR. Furthermore, the possible existence of isomiRs for miR-182 cannot be ruled out, since miRNA arrays demonstrated a 4-fold upregulation of miR-182 by ATO in PC3 cells, while Taqman qRT-PCR analysis detected an upregulation of only 1.5 to 2-fold.

The role of miR-182 in cancer development and progression remains incompletely defined, and may be tissue-specific; the literature contains what appear to be contradictory activities of miR-182 in different tissues. For example, the overexpression of miR-182 in prostate cancers [32],[33] and several other types of neoplasms [34] suggests a clear linkage to malignancy. By contrast, miR-182 suppresses cell proliferation in human gastric and lung adenocarcinoma cells [35],[36],[37] and *in vitro* invasion of prostate cancer cells [38], suggesting possible tumor suppressing activity in these tissues. In a previous study, we reported that the expression of miR-182 was stress-associated, and was linked to the inhibition of proliferation in MCF12F breast epithelial cells [29]. Our data are in general agreement with a report demonstrating that miR-182 upregulation is associated with stress-induced premature senescence in primary cultures of diploid fibroblast and trabecular meshwork cells [39].

In the present studies, both exposure to ATO and serum deprivation enhanced miR-182 expression in PC3 cells; this enhancement was p53-independent. These data differ from the results of a recent study in melanoma cells, in which doxorubicin induced a p53-dependent induction of miR-182 expression [20]. Although the molecular pathway(s) through which miR-182 expression is upregulated by ATO have not been identified conclusively, our results suggest that the inhibition of geranylgeranyl synthesis is a key factor; these data are consistent with the findings that ATO inhibits cell proliferation and induces autophagy by inhibiting geranylgeranyl biosynthesis, which is also consistent with a previous report [9], and suggest that geranylgeranylation of Rho family GTPases or Rab family GTPases may inhibit miR-182 expression. In this regard, Wang and colleagues [40] found that inhibition of isoprenylcysteine carboxymethyltransferase, an enzyme catalyzing methylation of prenylated small GTPases, induces autophagic cell death in PC3 cells. Rho GTPases have been shown to promote cancer cell growth, survival, and metastasis; inhibition of geranylgeranylation of Rho GTPases using highly selective inhibitors can block cancer cell growth and induce cell death [9]. Thus it is possible that inhibition of geranylgeranylation of Rho family GTPases may be critical for ATO to induce autophagy and enhance miR-182 expression.

Several target genes including MITF [20], FOXO1 [41] and RARγ [39] etc. have been identified as direct target genes of miR-182. We initially identified Bcl2 as a target of miR-182 by microarray analysis, strategy for which there is scientific precedent [42]. However, the use of microarray to identify miRNA targets is not always effective, since many miRNAs act to repress gene expression at the posttranscriptional level [43]. In our studies, knock-down of miR-182 had no effect on the expression of Bcl2 expression at the mRNA level, but increased the expression of Bcl2 protein. These data suggest that miR-182 regulates Bcl2 at posttranscriptional level by targeting the 3′UTR region, which has recently been demonstrated in melanoma cells [20].

When compared to controls, PC3 cell transfectants overexpressing miR-182 demonstrated a 26% reduction in Bcl2 mRNA, but no changes in the levels of Bcl2 protein. A possible explanation for this finding is that the basal expression level of Bcl2 protein is low, and the effect of miR-182 is physiologic (instead of pharmacologic). In such a situation, downregulation of the low basal levels of Bcl2 protein may not occur. By contrast, ATO significantly downregulated Bcl2 expression at both mRNA and protein levels. In consideration of the different activities of ATO and miR-182 transfectants, the effects of ATO on Bcl2 expression are presumably mediated through mechanisms other than upregulation of miR-182.

p21 is a key molecule in the regulation of the cell cycle and autophagy/cell death [12],[44]. It has been reported that the inhibition of cell proliferation by simvastatin is suppressed by silencing p21 [44], suggesting a possible role of p21 in statin action in cancer cells. The up-regulation of p21 expression by miR-182 may contribute to the effects of ATO on p21 expression; however, because less up-regulation of p21 was seen in miR-182 transfectants than in PC3 cells treated with ATO, other mechanisms must also be involved in ATO action in regulating p21. In this regard, ATO has been reported to increase p21 expression through inhibition of histone deacetylase (HDAC) activity and consequent induction of histone acetylation [45].

The present studies have identified miR-182 as a possible mediator of the antiproliferative and pro-autophagic activities of ATO in PC3 human prostate cancer cells. The link between up-regulation of miR-182, ATO inhibition of PC3 cell proliferation, and ATO induction of autophagy was clearly demonstrated by comparisons of ATO activity in PC3 versus LNCaP cells, and by comparisons of ATO activity in parental PC3 cells versus PC3 cell clones with different phenotypic responses. Confirmatory data were provided by studies conducted in PC3 cells transfected with an miR-182 mimic or an miR-182 inhibitor. Down-regulation of Bcl2 and/or up-regulation of p21 may act as downstream effectors of ATO action in these cells.

Clinically, the primary effect of statin treatment is the lowering of serum cholesterol; however, lowering of serum cholesterol and inhibition of prostate cancer cell growth appear to be independent events, although both may be mediated by effects on the mevalonate pathway. Our data suggest that inhibition of geranylgeranyl synthesis by atorvastatin plays a critical role in suppression of prostate cancer cell growth and induction of autophagy.

## Supporting Information

Figure S1 **Venn diagrams of microarray analyses of mRNA and miRNA expression in LNCaP and PC3 cells.** Cells were treated with 10 µM ATO for 24 h and total RNA was isolated and subjected to microarray analyses. (TIF)

Table S1 **Differentially expressed KEGG pathways identified in LNCaP cells exposed to atorvastatin.** LNCaP Cells were treated with 10 µM ATO for 24 h and total RNA was isolated and subjected to microarray analyses. Data was analyzed with Agilent Feature Extraction and GeneSpring GX v7.3.1 software packages (DOCX)

Table S2 **Differentially expressed KEGG pathways identified in PC3 cells exposed to atorvastatin.** PC3 Cells were treated with 10 µM ATO for 24 h and total RNA was isolated and subjected to microarray analyses. Data was analyzed with Agilent Feature Extraction and GeneSpring GX v7.3.1 software packages (DOCX)

Table S3 **miRNAs differentially regulated by ATO in PC3 cells following atorvastatin treatment (fold change cutoff value: 2.0).** PC3 Cells were treated with 10 µM ATO for 24 h and total RNA was isolated and subjected to miRNA microarray analyses. Data was analyzed with Agilent Feature Extraction and GeneSpring GX v7.3.1 software packages. (DOCX)

Table S4 **List of potential target genes of miR-182 in PC3 cells.** miR-182 target genes were selected on the basis of microarray dataset and predicted based on Targetscan and Pictar program analyses. (DOCX)
