# Supplementary figures and images for: Inhibition of Proliferation and Induction of Autophagy by Atorvastatin in PC3 Prostate Cancer Cells Correlate with Downregulation of Bcl2 and Upregulation of miR-182 and p21
Source: PLoS One. 2013 Aug 1;8(8):e70442. doi: 10.1371/journal.pone.0070442 (PMC3731278; doi:10.1371/journal.pone.0070442)

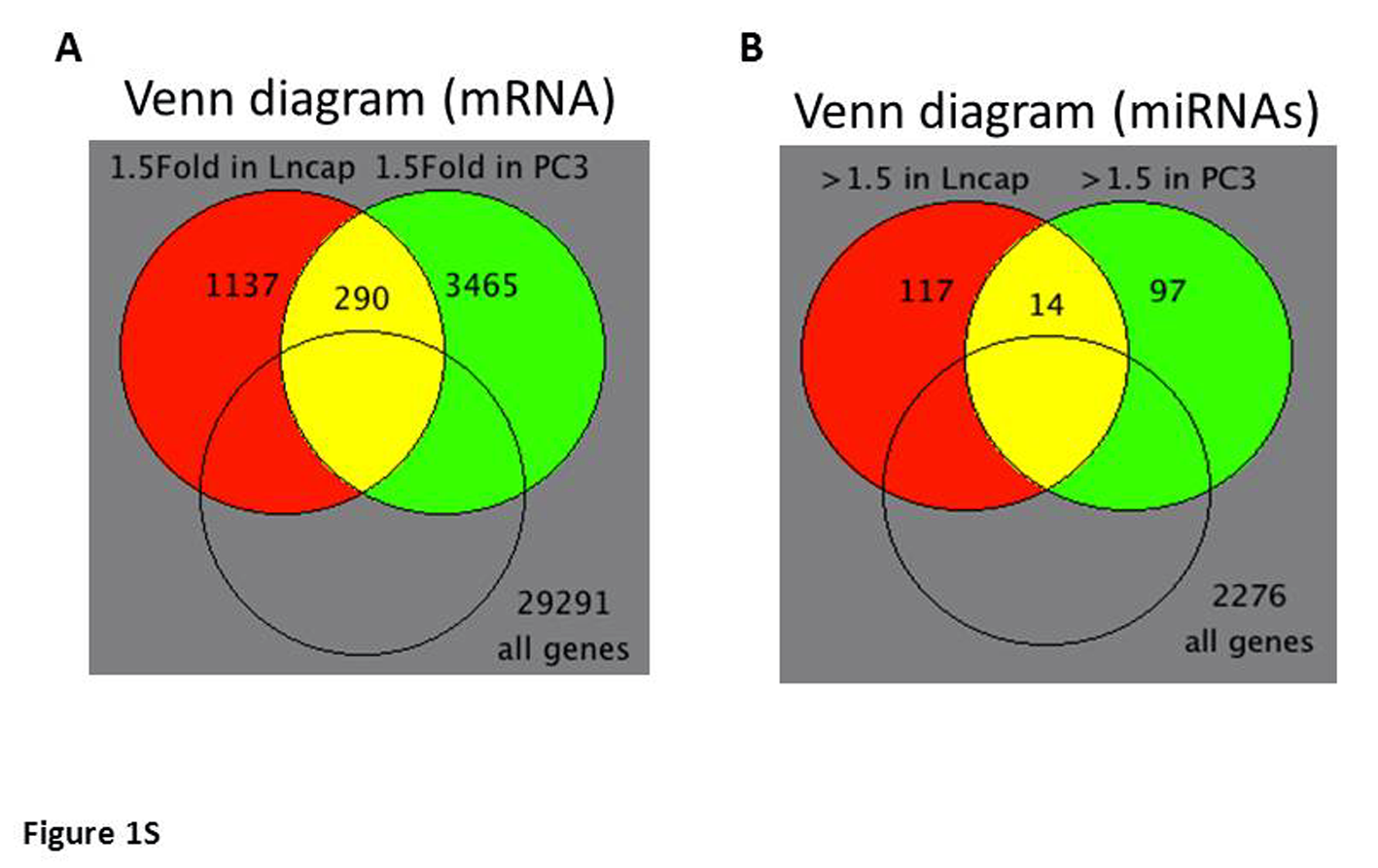

Supplement: Figure S1 — (TIF) [file pone.0070442.s001.tif]
