# Supplementary material for: Inhibition of Proliferation and Induction of Autophagy by Atorvastatin in PC3 Prostate Cancer Cells Correlate with Downregulation of Bcl2 and Upregulation of miR-182 and p21
Source: PLoS One. 2013 Aug 1;8(8):e70442. doi: 10.1371/journal.pone.0070442 (PMC3731278; doi:10.1371/journal.pone.0070442)
Supplement: Table S1 — (DOCX) [file pone.0070442.s002.docx]

**Table 1S. Differentially expressed KEGG pathways identified in LNCaP cells exposed to atorvastatin**

1. Steroid biosynthesis (*p* value of 1.30 x 10^-14^)

2. Cholesterol biosynthesis (*p* value of 2.10 x 10^-13^)

3. Sterol biosynthesis (*p* value of 2.46 x 10^-6^)

4. SREBP – control of lipid synthesis pathway (*p* value of 5.19 x 10^-4^)

5. Terpenoid biosynthesis (*p* value of 1.32 x 10^-3^)

6. Androgen and estrogen metabolism (*p* value of 6.60 x 10^-3^)

7. Pantothenate and CoA biosynthesis (*p* value of 9.30 x 10^-3^)
