# Supplementary material for: Inhibition of Proliferation and Induction of Autophagy by Atorvastatin in PC3 Prostate Cancer Cells Correlate with Downregulation of Bcl2 and Upregulation of miR-182 and p21
Source: PLoS One. 2013 Aug 1;8(8):e70442. doi: 10.1371/journal.pone.0070442 (PMC3731278; doi:10.1371/journal.pone.0070442)
Supplement: Table S2 — (DOCX) [file pone.0070442.s003.docx]

**Table S2. Differentially expressed KEGG pathways identified in PC3 cells exposed to atorvastatin**

1. DNA Replication *p* value of 2.04 x 10^-15^

2. G1 to S cell cycle control *p* value of 2.15 x 10^-14^

3. Cell cycle *p* value of 1.32 x 10^-8^

4. p53 signaling pathway *p* value of 9.05 x 10^-8^

5. Sonic_Hedgehog *p* value of 1.41 x 10^-6^

6. Phosphatidylinositol signaling system *p* value of 3.34 x 10^-6^

7. Small cell lung cancer *p* value of 3.69 x 10^-5^

8. Prostaglandin Synthesis and Regulation *p* value of 4.48 x 10^-5^

9. Nicotinate and nicotinamide metabolism *p* value of 2.27 x 10^-4^

10. Cholesterol Biosynthesis *p* value of 3.11 x 10^-4^

11. alpha-Linolenic acid metabolism *p* value of 3.33 x 10^-4^

12. Inositol phosphate metabolism *p* value of 3.51 x 10^-4^

13. Hypertrophy Model *p* value of 3.64 x 10^-4^

14. DNA polymerase *p* value of 3.95 x 10^-4^

15. Focal Adhesion *p* value of 6.40 x 10^-4^

16. Nicotinate and nicotinamide metabolism *p* value of 7.34 x 10^-4^

17. Cell Communication *p* value of 7.61 x 10^-4^

18. Small Ligand GPCRs *p* value of 1.14 x 10^-3^

19. Tryptophan metabolism *p* value of 1.20 x 10^-3^

20. Glycerophospholipid metabolism *p* value of 1.64 x 10^-3^

21. Ether lipid metabolism *p* value of 1.98 x 10^-3^

22. Fructose and mannose metabolism *p* value of 2.35 x 10^-3^

23. Focal adhesion *p* value of 2.40 x 10^-3^

24. Purine metabolism *p* value of 2.85 x 10^-3^

25. Pyrimidine metabolism *p* value of 3.30 x 10^-3^

26. Myometrial Relaxation and Contraction Pathways *p* value of 3.65 x 10^-3^

27. Sterol biosynthesis *p* value of 3.87 x 10^-3^

28. MAPK signaling pathway *p* value of 4.47 x 10^-3^

29. Bladder cancer *p* value of 4.76 x 10^-3^

30. Glycerolipid metabolism *p* value of 4.85 x 10^-3^

31. Biosynthesis of steroids *p* value of 4.88 x 10^-3^

32. Sphingoglycolipid metabolism *p* value of 5.56 x 10^-3^

33. Butanoate metabolism *p* value of 5.57 x 10^-3^

34. TGF-beta signaling pathway *p* value of 6.04 x 10^-3^

35. Limonene and pinene degradation *p* value of 6.14 x 10^-3^

36. Lysine degradation *p* value of 6.20 x 10^-3^

37. Linoleic acid metabolism *p* value of 6.62 x 10^-3^

38. Folate biosynthesis *p* value of 6.70 x 10^-3^

39. TNFR1_Signaling_Pathway_pathway *p* value of 6.73 x 10^-3^

40. TGF-beta Receptor Signaling Pathway *p* value of 6.90 x 10^-3^

41. Tetrachloroethene degradation *p* value of 7.06 x 10^-3^

42. GnRH signaling pathway *p* value of 7.50 x 10^-3^

43. Complement and coagulation cascades *p* value of 8.72 x 10^-3^

44. Complement Activation, Classical Pathway *p* value of 9.34 x 10^-3^

45. Triacylglyceride Synthesis *p* value of 9.40 x 10^-3^

46. Glioma - Homo sapiens *p* value of 9.49 x 10^-3^
