# Supplementary material for: Inhibition of Proliferation and Induction of Autophagy by Atorvastatin in PC3 Prostate Cancer Cells Correlate with Downregulation of Bcl2 and Upregulation of miR-182 and p21
Source: PLoS One. 2013 Aug 1;8(8):e70442. doi: 10.1371/journal.pone.0070442 (PMC3731278; doi:10.1371/journal.pone.0070442)
Supplement: Table S3 — (DOCX) [file pone.0070442.s004.docx]

**Table S3. miRNAs differentially regulated by ATO in PC3 cells following atorvastatin treatment (fold change cutoff value: 2.0).**

| **No.** | **LNCaP: ATO/CTRL** | **PC3: ATO/CTRL** | **GeneName** | **Gene id** |
| --- | --- | --- | --- | --- |
| 1 | 1.00 | 14.04 | hsa-miR-555 | MIMAT0003219 |
| 2 | 0.83 | 11.26 | hsa-miR-548d-5p | MIMAT0004812 |
| 3 | 1.00 | 10.05 | hsa-miR-654-5p | MIMAT0003330 |
| 4 | 1.00 | 9.11 | hsa-miR-935 | MIMAT0004978 |
| 5 | 1.00 | 8.47 | hsa-miR-487a | MIMAT0002178 |
| 6 | 1.00 | 7.85 | hsa-miR-409-5p | MIMAT0001638 |
| 7 | 1.00 | 7.10 | hsa-miR-661 | MIMAT0003324 |
| 8 | 1.09 | 6.97 | hsa-miR-190b | MIMAT0004929 |
| 9 | 1.00 | 6.66 | hsa-miR-583 | MIMAT0003248 |
| 10 | 1.00 | 6.61 | hsa-miR-331-5p | MIMAT0004700 |
| 11 | 1.00 | 6.32 | hsa-miR-448 | MIMAT0001532 |
| 12 | 1.00 | 6.26 | hsa-miR-760 | MIMAT0004957 |
| 13 | 1.00 | 6.23 | hsa-miR-770-5p | MIMAT0003948 |
| 14 | 1.00 | 6.16 | hsa-miR-583 | MIMAT0003248 |
| 15 | 1.00 | 5.22 | hsa-miR-224-5p | MIMAT0000281 |
| 16 | 1.00 | 5.22 | hsa-miR-553 | MIMAT0003216 |
| 17 | 1.00 | 5.05 | hsa-miR-1539 | MIMAT0007401 |
| 18 | 1.00 | 4.85 | hsa-miR-586 | \|MIMAT0003252 |
| 19 | 1.00 | 4.46 | hsa-miR-182-5p | MIMAT0000259 |
| 20 | 1.00 | 4.39 | hsa-miR-658 | MIMAT0003336 |
| 21 | 0.03 | 4.38 | hsa-miR-1827 | MIMAT0006767 |
| 22 | 1.00 | 4.35 | hsa-miR-601 | MIMAT0003269 |
| 23 | 0.57 | 4.25 | hsa-miR-500a-5p | MIMAT0004773 |
| 24 | 1.00 | 4.17 | hsa-miR-655 | MIMAT0003331 |
| 25 | 0.72 | 4.05 | hsa-miR-573 | MIMAT0003238 |
| 26 | 1.00 | 3.93 | hsa-miR-145-5p | MIMAT0000437 |
| 27 | 0.70 | 3.01 | hsa-miR-500a-5p | MIMAT0004773 |
| 28 | 0.62 | 2.96 | hsa-miR-892b | MIMAT0004918 |
| 29 | 0.79 | 2.70 | hsa-miR-146b-5p | MIMAT0002809 |
| 30 | 0.10 | 2.27 | hsa-miR-194-5p | MIMAT0000460 |
| 31 | 2.61 | 0.50 | hsa-miR-494 | MIMAT0002816 |
| 32 | 1.00 | 0.21 | hsa-miR-651 | MIMAT0003321 |
| 33 | 1.00 | 0.21 | hsa-miR-647 | MIMAT0003317 |
| 34 | 1.00 | 0.20 | hsa-miR-539-5p | MIMAT0003163\| |
| 35 | 1.00 | 0.20 | hsa-miR-1267 | MIMAT0005921 |
| 36 | 1.00 | 0.16 | hsa-miR-1227 | MIMAT0005580 |
| 37 | 0.07 | 0.16 | hsa-miR-1255a | MIMAT0005906 |
| 38 | 1.00 | 0.15 | hsa-miR-147a | MIMAT0000251 |
| 39 | 1.00 | 0.09 | hsa-miR-550a-5p | MIMAT0004800 |
| 40 | 1.00 | 0.09 | hsa-miR-549 | MIMAT0003333 |
| 41 | 1.00 | 0.05 | hsa-miR-892a | MIMAT0004907 |
| 42 | 1.00 | 0.05 | hsa-miR-323b-5p | MIMAT0001630 |
