# Supplementary material for: Inhibition of Proliferation and Induction of Autophagy by Atorvastatin in PC3 Prostate Cancer Cells Correlate with Downregulation of Bcl2 and Upregulation of miR-182 and p21
Source: PLoS One. 2013 Aug 1;8(8):e70442. doi: 10.1371/journal.pone.0070442 (PMC3731278; doi:10.1371/journal.pone.0070442)
Supplement: Table S4 — (DOCX) [file pone.0070442.s005.docx]

**Table S4. List of potential target genes of miR-182 in PC3 cells**

| up-regulation | | | down-regulation | | |
| --- | --- | --- | --- | --- | --- |
| Target gene | Accession No. | Ato/Ctrl ratio | Target gene | Accession No. | Ato/Ctrl ratio |
| FOXF2 | NM_001452 | 4.48 | BCL2 | NM_000633 | 0.14 |
| SLC1A1 | NM_004170 | 1.74 | RFTN1 | NM_015150 | 0.6 |
| ACVR1 | NM_001105 | 1.58 | BNC2 | NM_017637 | 0.5 |
| SH3BGRL3 | NM_031286 | 1.57 | TBX1 | NM_080647 | 0.56 |
| KLF7 | NM_003709 | 4.13 | SH3BP4 | NM_014521 | 0.59 |
| PRDM1 | NM_001198 | 1.65 | IGF1R | NM_000875 | 0.63 |
| ZFP36 | NM_003407 | 2.08 | MCMBP | NM_024834 | 0.57 |
| CCDC92 | NM_025140 | 1.58 | ZNF280B | NM_080764 | 0.55 |
| FOXQ1 | NM_033260 | 1.55 | RAB23 | NM_016277 | 0.64 |
| HOOK3 | NM_032410 | 1.74 | FRMD4A | NM_018027 | 0.4 |
| VAT1 | NM_006373 | 1.54 | ARL4C | NM_005737 | 0.59 |
| TMEM50B | NM_006134 | 1.95 | ELL | NM_006532 | 0.37 |
| GK | NM_000167 | 2.1 | LPHN2 | NM_012302 | 0.59 |
| MAP1LC3B | NM_022818 | 1.52 | ELL2 | NM_012081 | 0.62 |
| CELSR1 | NM_014246 | 1.53 | CHST10 | NM_004854 | 0.59 |
| INSIG1 | NM_005542 | 2.24 | AMOTL2 | NM_016201 | 0.28 |
| KLF13 | NM_015995 | 2.44 | EIF2C1 | NM_012199 | 0.64 |
| BHLHE41 | NM_030762 | 1.54 |  |  |  |
| PPP1R2 | NM_006241 | 1.57 |  |  |  |
| YWHAG | NM_012479 | 1.64 |  |  |  |
| SHC4 | NM_203349 | 2.1 |  |  |  |
| ANTXR2 | NM_058172 | 1.54 |  |  |  |
| TP53INP2 | NM_021202 | 1.56 |  |  |  |
| ATXN1 | NM_000332 | 3.63 |  |  |  |
